# Circ_MDM2_000139, Circ_ATF2_001418, Circ_CDC25C_002079, and Circ_BIRC6_001271 Are Involved in the Functions of XAV939 in Non-Small Cell Lung Cancer

**DOI:** 10.1155/2019/9107806

**Published:** 2019-11-27

**Authors:** Haixiang Yu, Lei Xu, Zhengjia Liu, Bo Guo, Zhifeng Han, Hua Xin

**Affiliations:** Department of Thoracic Surgery, China-Japan Union Hospital of Jilin University, Changchun, Jilin Province 130033, China

## Abstract

**Background:**

The small molecule inhibitor XAV939 could inhibit the proliferation and promote the apoptosis of non-small cell lung cancer (**NSCLC**) cells. This study was conducted to identify the key circular RNAs (circRNAs) and microRNAs (miRNAs) in XAV939-treated NSCLC cells.

**Methods:**

After grouping, the NCL-H1299 cells in the treatment group were treated by 10 *μ*M XAV939 for 12 h. RNA-sequencing was performed, and then the differentially expressed circRNAs (DE-circRNAs) were analyzed by the edgeR package. Using the clusterprofiler package, enrichment analysis for the hosting genes of the DE-circRNAs was performed. Using Cytoscape software, the miRNA-circRNA regulatory network was built for the disease-associated miRNAs and the DE-circRNAs. The DE-circRNAs that could translate into proteins were predicted using circBank database and IRESfinder tool. Finally, the transcription factor (TF)-circRNA regulatory network was built by Cytoscape software. In addition, A549 and HCC-827 cell treatment with XAV939 were used to verify the relative expression levels of key DE-circRNAs.

**Results:**

There were 106 DE-circRNAs (including 61 upregulated circRNAs and 45 downregulated circRNAs) between treatment and control groups. Enrichment analysis for the hosting genes of the DE-circRNAs showed that *ATF2* was enriched in the TNF signaling pathway. Disease association analysis indicated that 8 circRNAs (including circ_MDM2_000139, circ_ATF2_001418, circ_CDC25C_002079, and circ_BIRC6_001271) were correlated with NSCLC. In the miRNA-circRNA regulatory network, let-7 family members⟶circ_MDM2_000139, miR-16-5p/miR-134-5p⟶circ_ATF2_001418, miR-133b⟶circ_BIRC6_001271, and miR-221-3p/miR-222-3p⟶circ_CDC25C_002079 regulatory pairs were involved. A total of 47 DE-circRNAs could translate into proteins. Additionally, circ_MDM2_000139 was targeted by the TF *POLR2A*. The verification test showed that the relative expression levels of circ_MDM2_000139, circ_CDC25C_002079, circ_ATF2_001418, and circ_DICER1_000834 in A549 and HCC-827 cell treatment with XAV939 were downregulated comparing with the control.

**Conclusions:**

Let-7 family members and *POLR2A* targeting circ_MDM2_000139, miR-16-5p/miR-134-5p targeting circ_ATF2_001418, miR-133b targeting circ_BIRC6_001271, and miR-221-3p/miR-222-3p targeting circ_CDC25C_002079 might be related to the mechanism in the treatment of NSCLC by XAV939.

## 1. Introduction

In lung cancers, non-small cell lung cancer (NSCLC) and small-cell lung carcinoma (SCLC) are the two main types [1]. Lung cancer usually can result in shortness of breath, coughing, chest pains, and weight loss [2, 3]. In 2012, there were 1.8 million new cases of lung cancer and led to 1.6 million deaths globally [4]. Especially, NSCLC takes up 85% of all lung cancer cases, which are mainly induced by smoking [5]. As NSCLC progresses from stage I to stage IV, the five-year survival rate reduces from 47% to 1% [6]. Therefore, it is essential to study the treatment for NSCLC and related mechanism.

XAV939 is a tankyrase (TNKS) inhibitor and an indirect Wnt/*β*-catenin signaling inhibitor that is often used to inhibit proliferation of NSCLC cells. Guo et al. reported that XAV939 could inhibit the viability of SCLC NCI–H446 cells by causing cell apoptosis through the Wnt signaling pathway [7]. Besides, XAV939 also repressed the proliferation and migration of lung adenocarcinoma A549 cells through attenuating the Wnt signaling pathway [8]. Moreover, it is known that circular RNAs (circRNAs) are implicated in the development and progression of cancers [9]. CircRNA_100876 is tightly correlated with the oncogenesis of NSCLC, which may function as a promising prognostic marker and therapeutic target for the disease [10]. Circ_0014130 can serve as a candidate biomarker for NSCLC, and may play critical roles in the formation of the tumor [11]. Besides, plenty of reports have stated that the circRNAs usually be involved in the NSCLC by the interaction with miRNAs. CircRNA forkhead box O3 (FOXO3) acts as a tumor suppressor via sponging miR-155 in NSCLC, and thus expression restoration of circRNA FOXO3 can be a new therapeutic option for NSCLC [12]. CircRNA_100833 (also called circRNA fatty acid desaturases 2, circFADS2) promotes the progression of NSCLC through mediating miR-498 expression; therefore, circFADS2 can be utilized as a novel target for treating NSCLC [13]. However, the key circRNAs associated with the pathogenesis of NSCLC have not been entirely revealed.

Our preliminary experiments showed that XAV939 could significantly inhibit the proliferation and promote the apoptosis of NSCLC NCL-H1299 cells, and 10 *μ*M is the appropriate XAV939 concentration for treating NCL-H1299 cells (data not shown). In this study, XAV939 (10 *μ*M) was used to treat NCL-H1299 cells in the treatment group. After high throughput sequencing, the sequencing data were analyzed using various bioinformatics methods. This study might contribute to revealing the key circRNAs mediated by XAV939 in NCL-H1299 cells.

## 2. Materials and Methods

### 2.1. Sample Source

The NSCLC NCL-H1299 cell line was obtained from the Type Culture Collection of the Chinese Academy of Sciences (Shanghai, China). Six NCL-H1299 cell samples were randomly and evenly divided into the treatment group and control group. The cells in the treatment group were treated by XAV939 (10 *μ*M) for 12 h. The cells in the control group were treated by equal volume of dimethyl sulfoxide (DMSO). Afterwards, the cells were harvested for the following sequencing.

### 2.2. Library Construction and RNA-Sequencing

Using the TRIzol reagent (Takara Biotechnology Co., Ltd., Dalian, China), total RNA was extracted from the cells according to the manufacturer's instruction. Then, the quality and quantity of total RNA were detected using a Thermo Scientific NanoDrop 2000 (Thermo Fisher Scientific, Inc., Wilmington, DE, USA). Subsequently, cDNA library was constructed following the manufacturer's manual by a TruseqTM RNA Library Prep kit for Illumina® (cat no. E7530L; New England BioLabs, Inc., Ipswich, MA, USA). Based on the 150 paired end method [14], sequencing was performed by the Illumina Hiseq 4000 platform (Illumina, Inc., San Diego, CA, USA). The sequencing data were deposited in Sequence Read Archives (SRA, https://http://www.ncbi.nlm.nih.gov/sra) database under the accession number SRP136747.

### 2.3. Quality Control and Preprocessing of Sequencing Data

Quality control of the sequencing data was conducted using Trimmomatic tool [15]. Barcode sequences were removed, and the bases with continuous quality <10 at two ends of the reads were taken out. Subsequently, the reads that contained less than 80% bases with *Q* > 20 were filtered out, and the reads with length <50 nt were also wiped off. Based on TopHat2 software (http://ccb.jhu.edu/software/tophat) [16], clean reads were aligned to the human reference genome (GRCh38.p7 and GENCODE) [17]. In order to obtain back-spliced junctions reads, the reads that could not be compared to the reference genome in a linear way were compared in a nonlinear way using TopHat-fusion algorithm [18].

### 2.4. Identification and Annotation of CircRNAs

CircRNAs were identified using CIRCexplorer2 software [19], and the circRNAs with junctions read count > 2 were selected. Based on the locations of circRNAs in the genome and their relationships with genes, the selected circRNAs were annotated. Firstly, the circRNAs were classified according to their locations. Secondly, the circRNAs were conducted with functional annotation based on the circRNA-hosting genes. The RefSeq gene annotation files downloaded from the University of California Santa Cruz (UCSC, http://genome.ucsc.edu/) database [20] were used as references for annotating. Finally, the circRNAs were named combined with the names of their hosting genes.

### 2.5. Identification of the Differentially Expressed CircRNAs (DE-CircRNAs) and Enrichment Analysis

The expression of the circRNAs was estimated based on the number of back-spliced reads. Using the edgeR package (http://bioconductor.org/packages/release/bioc/html/edgeR.html) in R [21], the DE-circRNAs between treatment and control groups were screened. The |log_2_ fold change (FC)| > 0.585 and *p* value <0.05 were defined as the thresholds. The expression of circRNA was required to be higher than 0 in at least 2 samples, and the ineligible circRNAs were filtered out.

Using the clusterprofiler package (http://bioconductor.org/packages/release/bioc/html/clusterProfiler.html) in R [22], the hosting genes of the DE-circRNAs were studied with Gene Ontology (GO, including biological process (BP), molecular function (MF), and cellular component (CC) categories) [23] and Kyoto encyclopedia of genes and genomes (KEGG) [24] enrichment analyses. The significant threshold was set at *p* value <0.05.

### 2.6. MiRNA Sponge Analysis and Disease Association Analysis

Previous studies have found that there are multiple target sites of miRNAs in some circRNAs sequences, and thus circRNAs can bind with miRNAs to play certain regulatory roles in vivo [25, 26]. Using miRanda tool [27], miRNA-circRNA pairs were predicted for the DE-circRNAs.

Based on DisGenet (http://www.disgenet.org) [28] and miRWalk (http://mirwalk.uni-hd.de/) [29] databases, the genes or miRNAs correlated with NSCLC were searched. If the hosting genes of DE-circRNAs were related to NSCLC, the DE-circRNAs were deemed to be associated with the disease. For the disease-associated miRNAs and the DE-circRNAs, the miRNA-circRNA regulatory network was built using Cytoscape software (http://www.cytoscape.org) [30].

### 2.7. Prediction of the CircRNAs with the Ability to Translate into Proteins

The corresponding data of the DE-circRNAs were obtained from circBank (http://www.circbank.cn/) and circBase [31] databases. The DE-circRNAs with protein-encoding ability (coding_prob > 0.364) were selected from circBank database. Besides, the IRESfinder tool [32] was used to predict whether there were internal ribosome entry sites (IRESs) in the DE-circRNAs. The circRNAs with both protein-encoding ability and IRESs were considered to be with the ability to translate into proteins.

### 2.8. Construction of Transcription Factor (TF)-CircRNA Regulatory Network

The TRCirc database [33] (http://www.licpathway.net/TRCirc/view/index) integrates the chip-sequencing data, RNA-sequencing data, and 450k array data in ENCODE database and combines with human circRNA information in circBase database for the analysis of circRNA transcriptional regulation. TFs were predicted for the DE-circRNAs using TRCirc database [33], and then TF-circRNA regulatory network was constructed using Cytoscape software [30].

### 2.9. Validation of Key DE-circRNAs in A549 and HCC-827 Cell Treatment with XAV939

In order to observe the effect of XAV939 on the expressions of key DE-circRNAs, A549 and HCC-827 cells were used. A549 and HCC-827 cells were purchased from the Cell Bank of Chinese Academy of Science (Shanghai, China). Cells in the logarithmic growth phase of the experimental group were treated with 10 *μ*M XAV939, and the control group was supplemented with an equal volume of DMSO. Then, the total RNA was extracted by the TRIzol reagent (Takara Biotechnology Co., Ltd., Dalian, China). Finally, the relative expression levels of key DE-circRNAs were detected by the real-time reverse transcription polymerase chain reaction (RT-PCR). The primer information is shown in the Supplementary [Supplementary-material supplementary-material-1].

## 3. Results

### 3.1. Data Preprocessing, Identification, and Annotation of CircRNAs

**Figure 1:**
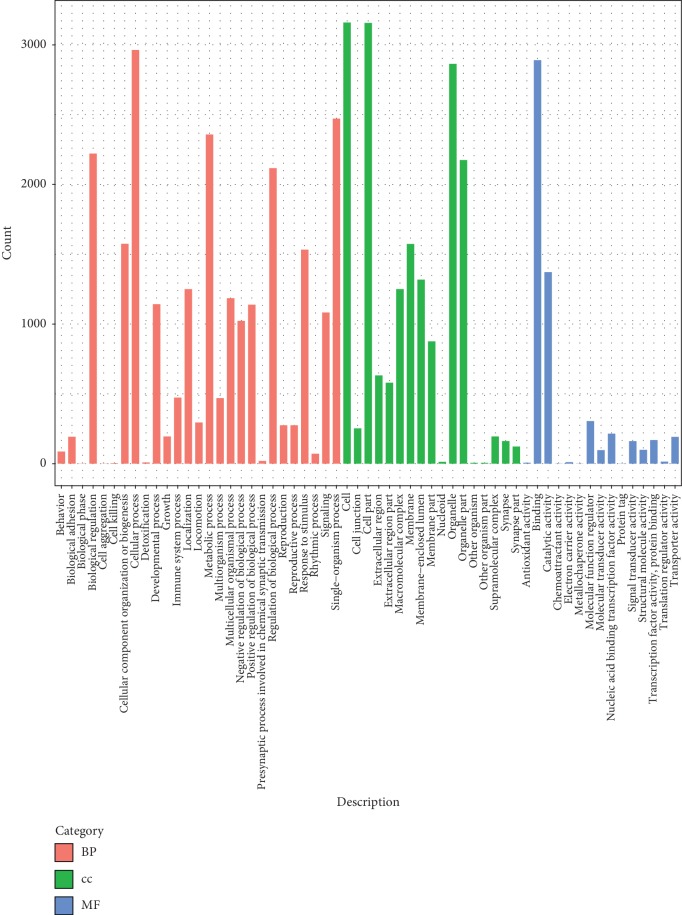
The functional terms enriched for the hosting genes of the identified circular RNAs (circRNAs). BP, biological process; CC, cellular component; and MF, molecular function.

The results of quality control and sequence alignment separately were listed in Tables 1 and 2. A total of 8914 circRNAs corresponding to 3542 hosting genes were identified. After annotation of the circRNAs, the GO functional terms enriched for the hosting genes of the circRNAs are shown in Figure 1, such as biological regulation, cellular progress, chemoattractant activity, and so on.

### 3.2. Differential Expression Analysis and Enrichment Analysis

**Figure 2:**
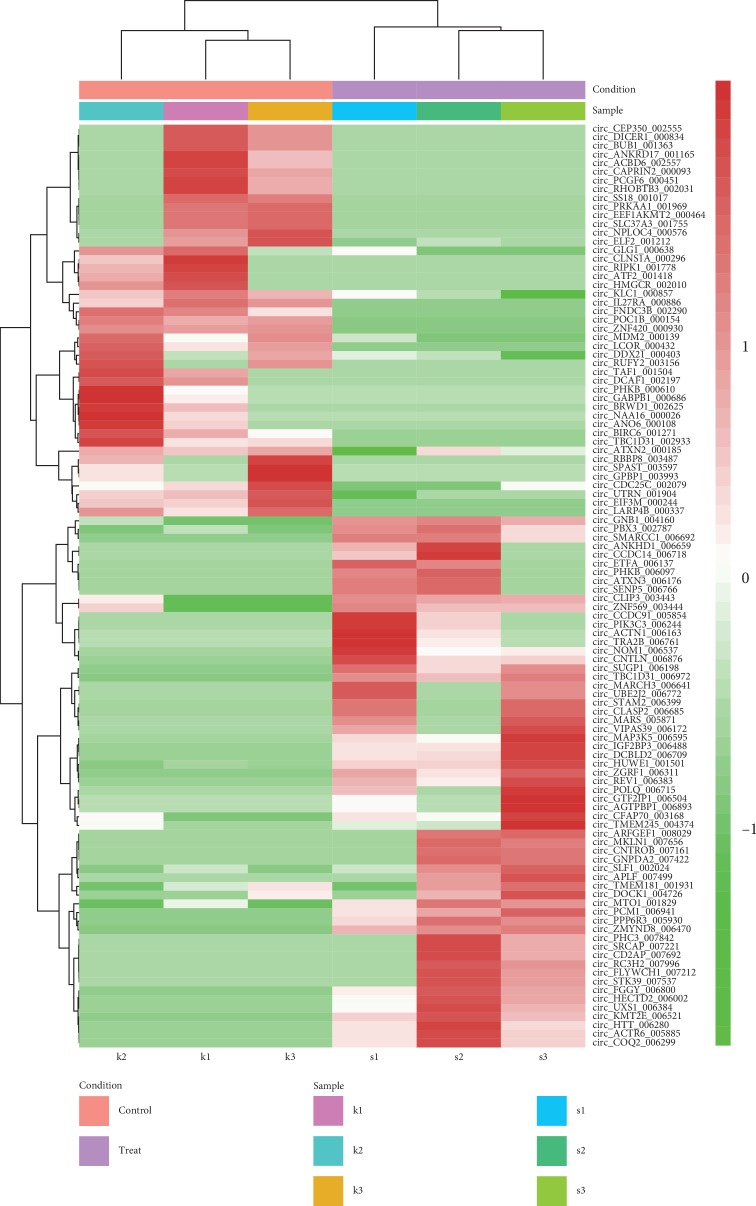
The clustering heatmap of the differentially expressed circular RNAs (circRNAs). s1, s2, and s3 represent the samples in the treatment group. k1, k2, and k3 represent the samples in the control group.

**Figure 3:**
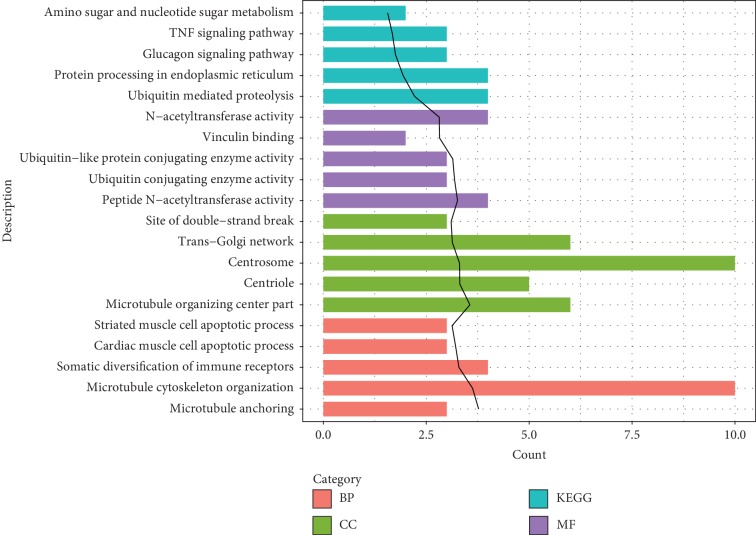
The functional terms and pathways enriched for the hosting genes of the differentially expressed circular RNAs (circRNAs) (top 5 listed). BP, biological process; CC, cellular component; MF, molecular function; and KEGG, Kyoto encyclopedia of genes and genomes. The black trend line represents -log10 (*p* value).

There were 106 DE-circRNAs between treatment and control groups, including 61 upregulated circRNAs and 45 downregulated circRNAs (Table 3). The clustering heatmap of the DE-circRNAs is presented in Figure 2. The samples of control and treatment groups were significantly separated by the DE-circRNAs. For the hosting genes of the DE-circRNAs, 204 BP terms (such as microtubule cytoskeleton organization), 27 CC terms (such as centrosome), 40 MF terms (such as N-acetyltransferase activity), and 9 KEGG pathways (such as the TNF signaling pathway, which involved activating transcription factor 2 (*ATF2*)) were enriched (Figure 3).

### 3.3. miRNA Sponge Analysis and Disease Association Analysis

**Figure 4:**
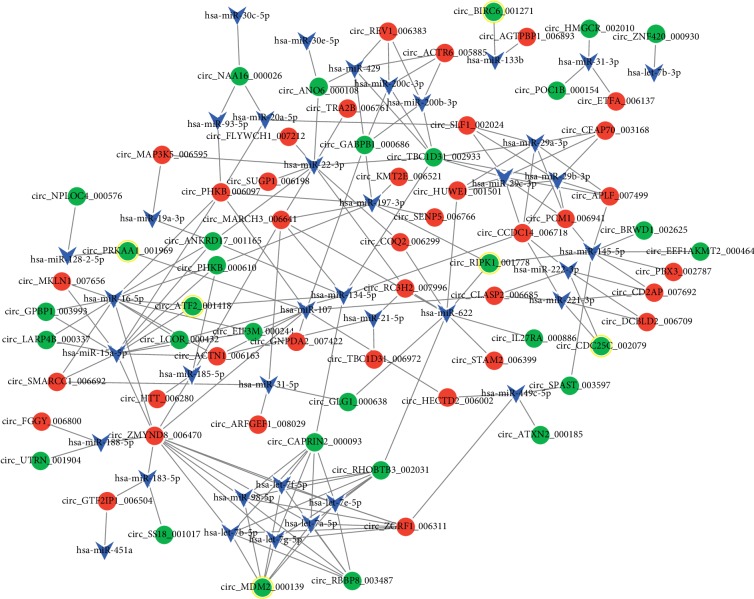
The miRNA-circular RNA (circRNA) regulatory network. Blue triangles, red circles, and green circles represent miRNAs, upregulated circRNAs, and downregulated circRNAs, respectively. The circles with yellow ring represent disease-associated circRNAs.

After miRNA-circRNA pairs were predicted for the DE-circRNAs, the top 10 miRNAs targeting more circRNAs were selected and listed in Table 4. Through disease association analysis, 8 circRNAs (including circ_MDM2_000139, corresponding to hosting gene *MDM2* (murine double minute 2); circ_ATF2_001418, corresponding to hosting gene *ATF2*; circ_CDC25C_002079, corresponding to hosting gene *CDC25C* (cell division cycle 25C); and circ_BIRC6_001271, corresponding to hosting gene *BIRC6* (baculoviral inhibitor of apoptosis repeat-containing 6)) were found to be related to the disease (Table 5). Finally, the miRNA-circRNA regulatory network was constructed, which had 106 nodes (including 38 miRNAs and 64 circRNAs) and 194 regulatory pairs (including let-7 family members⟶circ_MDM2_000139, miR-16-5p/miR-134-5p⟶circ_ATF2_001418, miR-133b⟶circ_BIRC6_001271, hsa-miR-197-3p circ_RIPK1_001778, hsa-miR-128-2-5p circ_PRKAA1_001969, and miR-221-3p/miR-222-3p⟶circ_CDC25C_002079) (Figure 4).

### 3.4. Prediction of the CircRNAs with the Ability to Translate into Proteins

The DE-circRNAs were mapped to circBank and circBase databases, and three novel circRNAs (including circ_ATF2_001418, circ_FLYWCH1_007212, and circ_GTF2IP1_006504) were not found in the two databases. Among the 65 DE-circRNAs with protein-encoding ability, there were 47 DE-circRNAs which had IRESs. Therefore, the 47 DE-circRNAs were taken as circRNAs that could translate into proteins.

### 3.5. Construction of TF-CircRNA Regulatory Network

**Figure 5:**
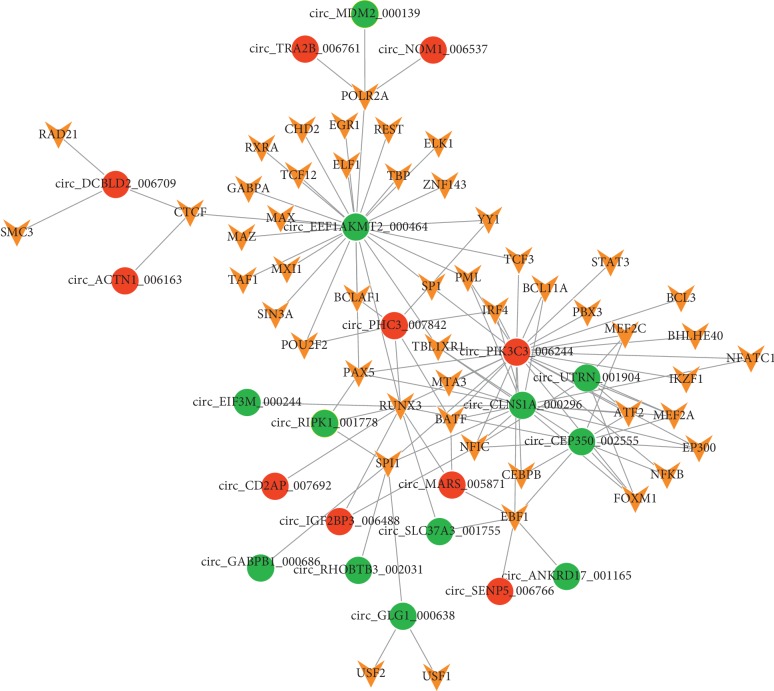
The transcription factor (TF)-circRNA regulatory network. Orange inverted triangles represent TFs. Red circles and green circles represent upregulated circRNAs and downregulated circRNAs, respectively. The circles with yellow ring represent the disease-associated circRNAs.

After TFs were predicted for the DE-circRNAs, the TF-circRNA regulatory network was built (Figure 5). In the TF-circRNA regulatory network, there were 72 nodes (including 22 circRNAs and 50 TFs) and 115 edges. Especially, circ_MDM2_000139, which was correlated with the disease, was targeted by RNA polymerase II (*POLR2A*) in the TF-circRNA regulatory network.

The expressed levels of key DE-circRNAs in A549 and HCC-827 cells are treated by XAV939

**Figure 6:**
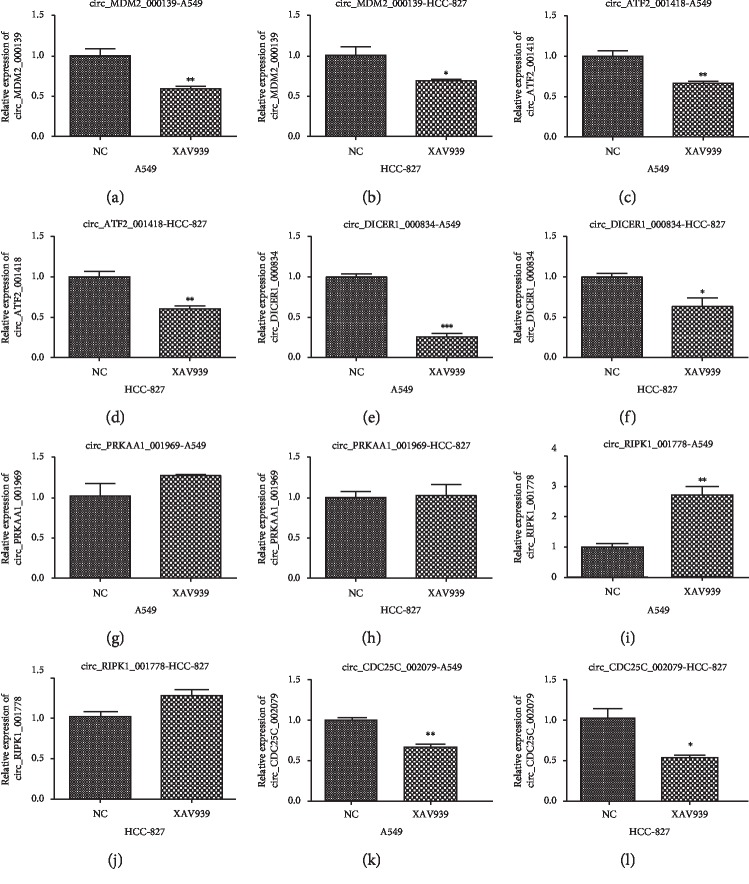
The relative expressions of key DE-circRNAs in the A549 and HCC-827 cells. NC represents the control cells. XAV939 represents the A549 and HCC-827 cells treated by 10 *μ*M XAV939. ^*∗*^*P* < 0.05 indicates a significant difference compared with that of the NC group; ^*∗∗*^*P* < 0.01 indicates a very significant difference compared with that of the NC group; ^*∗∗∗*^*P* < 0.001.

In order to validate the effect of XAV939 on other NSCLC cells, the relative levels of key DE-cirRNAs such as circ_MD-M2_000139, circ_ATF2_001418, circ_DICER1_000834, circ_PRKAA1_001969, circ_RIPK1_001778, and circ_CDC25C_002079 were further studied in A549 and HCC-827 cells. As shown in Figure 6, comparing with the NC group, the relative expression levels of circ_MDM2_000139, circ_CDC25C_002079, circ_ATF2_001418, and circ_DICER1_000834 in A549 and HCC-827 cell treatment with XAV939 were downregulated (*P* < 0.05 or *P* < 0.01). There were no differences in the circ_PRKAA1_001969 levels between the control and XAV939 group. In addition, after treatment with XAV939, the circ_RIPK1_001778 levels were upregulated in the A549 cells, and no obvious change was found in HCC-827 cells.

## 4. Discussion

In this study, 106 DE-circRNAs (including 61 upregulated circRNAs and 45 downregulated circRNAs) were screened between the treatment and control groups. Disease association analysis showed that 8 circRNAs (including circ_MDM2_000139, circ_ATF2_001418, circ_CDC25C_002079, and circ_BIRC6_001271) were correlated with NSCLC. Besides, let-7 family members⟶circ_MDM2_000139, miR-16-5p/miR-134-5p⟶circ_ATF2_001418, miR-133b⟶circ_BIRC6_001271, and miR-221-3p/miR-222-3p⟶circ_CDC25C_002079 regulatory pairs were involved in the miRNA-circRNA regulatory network. Furthermore, 47 DE-circRNAs were taken as circRNAs that could translate into proteins. In addition, circ_MDM2_000139 was found to be targeted by *POLR2A* in the TF-circRNA regulatory network. The results of validation experiments showed that circ_MDM2_000139, circ_CDC25C_002079, circ_ATF2_001418, and circ_DICER1_000834 were also downregulated in the A549 and HCC-827 cells after treatment with the XAV939, which were consistent with the sequencing results.

Tumor necrosis factor-*α* (*TNF-α*) acts as a critical inflammatory factor that links inflammation and tumor, which is also correlated with angiogenesis, proliferation, invasion, and migration in human cancers [34]. Via TNF-*α*/nuclear factor kappa B (NF-κB) and phosphatidylinositol 3-kinase (PI3K)/AKT pathways, sotetsuflavone suppresses the migration and invasion of NSCLC cells and may be effective in treating the tumor [35]. Through targeting *ATF2*, tumor suppressor miR-204 can inhibit cell proliferation and migration, and promote G1 arrest and cell apoptosis in NSCLC [36]. Elevated miR-16 is an independent factor that predicts unfavorable overall survival (OS) and disease-free survival (DFS), and thus miR-16 expression may be taken as a prognostic indicator in NSCLC [37]. Via mediating oncogenic cyclin D1 (*CCND1*), miR-134 represses proliferation, invasion, and migration and accelerates apoptosis of NSCLC cells [38, 39]. *ATF2* was enriched in the TNF signaling pathway, suggesting that miR-16-5p/miR-134-5p targeting circ_ATF2_001418 might act in the mechanisms of NSCLC through the TNF signaling pathway. We concluded that the TNF signaling pathway might be another potential target of XAV939.

*MDM2* and matrix metalloproteinase 9 (*MMP9*) expressions are related to the formation and migration of lung cancer; therefore, they can serve as the markers for the treatment and prognosis of the disease [40]. *MDM2* gene amplification is closely associated with DFS and OS, indicating that *MDM2* amplification can be used for predicting the survival of NSCLC patients who experienced surgical treatment [41]. The let-7 family members function as tumor suppressors in lung cancer, which can be repressed by Lin-28 and inhibit cell proliferation [42]. Through suppressing the transcription of *POLR2A*, the type II glycoprotein *CD26* plays an inhibitory role in tumor growth [43]. Therefore, let-7 family members and *POLR2A* targeting circ_MDM2_000139 might be also correlated with the progression of NSCLC. However, after consulting the literature, there were few reports providing direct relationship between let-7 family members, *POLR2A*, and XAV939.

Increased BIRC6 protein level may be a predictive factor for chemoresistance and an adverse prognostic marker for NSCLC, and inhibiting *BIRC6* may be a useful method for treating the tumor [44]. MiR-133b is found to be able to decrease cisplatin resistance, and its overexpression suppresses cell growth and invasion in cisplatin-resistant NSCLC via regulating glutathione-S-transferase P1 (*GSTP1*) [45]. Through reducing CDC25C and CDC2 protein levels, the heat shock protein 90 (*HSP90*) inhibitor is implicated in antiproliferative activity and tumor progression in lung cancer cells and thus can be applied for the treatment of lung cancer [46]. MiR-221 and miR-222 are involved in multiple human cancers, which play tumor-suppressive roles in lung cancer and may be promising targets for the therapy of the disease [47]. These indicated that miR-133b targeting circ_BIRC6_001271 and miR-221-3p/miR-222-3p targeting circ_CDC25C_002079 might be implicated in the pathogenesis of NSCLC. Dicer is important for microRNA-mediated silencing and other RNA interference, which were profoundly involved in cancer related networks [48]. Díaz-García et al. found that the DICER1 expression level varied among cancer specimens and 66% cancer samples had decreased DICER1 mRNA. Besides, the median overall survival (OS) of those with low level of DICER1 mRNA was substantially reduced [49]. In addition, the copy number variation of DICER1 correlates well with the expression and survival of NSCLC, and the increased expression DICER1 increases the survival [50]. In our study, we found that circ_DICER1_000834 was downregulated in the A549 and HCC-827 cells after treatment with the XAV939. The reports indicated that the DICER1 in the pathogenesis of NSCLC and circ_DICER1_000834 might play an important during the XAV939 treatment for NSCLC.

In conclusion, 106 DE-circRNAs between the treatment and control groups were identified. Besides, let-7 family members and *POLR2A* targeting circ_MDM2_000139, miR-16-5p/miR-134-5p targeting circ_ATF2_001418, miR-133b targeting circ_BIRC6_001271, and miR-221-3p/miR-222-3p targeting circ_CDC25C_002079 might be involved in the function during the treatment of NSCLC by XAV939. However, the roles of these RNAs and regulatory relationships in treatment of NSCLC by XAV939 needed to be further confirmed by experimental research studies.

## Figures and Tables

**Table 1:** The results of quality control for the sequencing data.

Sample	Raw reads	Clean reads	Effective rate (%)	Error rate (%)	Q20 (%)	Q30 (%)	GC content (%)
k3	55388409	54435729	98.28	0.01	98.17	95.44	46.97
k2	52553686	51681295	98.34	0.01	97.93	94.92	47.2
k1	52146382	51484123	98.73	0.01	98.18	95.44	47.73
s3	54313514	53504243	98.51	0.01	96.92	92.81	47.05
s2	49367345	48641646	98.53	0.01	98.19	95.47	47.52
s1	53737400	52909845	98.46	0.01	97.76	94.55	47.31

*Note.* s1, s2, and s3 represent the samples in the treatment group. k1, k2, and k3 represent the samples in the control group. Sample, the name of samples; raw reads, the number of raw reads; clean reads, the number of clean reads; error rate, the average base sequencing error rate; Q20, the percentage of the bases with Phred value > 20; Q30, the percentage of the bases with Phred value > 30; GC content, the percentage of G/C bases.

**Table 2:** The results of sequence alignment.

Reads	Mapping	k3	k2	k1	s3	s2	s1
Left reads	Input	54435729	51681295	51484123	53504243	48641646	52909845
	Mapped reads	43706641	41399669	40805316	42960786	38202327	41965536
	Mapped rate (%)	80.29	80.11	79.26	80.29	78.54	79.32
	Uniquely mapped	40483797	38479400	37712691	40029424	35183009	38751263
	Uniquely mapped rate (%)	74.37	74.46	73.25	74.82	72.33	73.24
Right reads	Input	54435729	51681295	51484123	53504243	48641646	52909845
	Mapped reads	41882371	39205669	39063079	40162292	36529153	39526360
	Mapped rate (%)	76.94	75.86	75.87	75.06	75.10	74.71
	Uniquely mapped	38760420	36422877	36072263	37394997	33623734	36501173
	Uniquely mapped rate (%)	71.20	70.48	70.06	69.89	69.13	68.99
Overall read	Mapped rate (%)	78.60	72.60	77.60	77.70	76.80	77.00

*Note.* s1, s2, and s3 represent the samples in the treatment group. k1, k2, and k3 represent the samples in the control group. Left/right reads, sequences at the two ends; input, the total number of sequences; mapped reads, the number of the reads mapped to the genome; mapping rate, the ratio of the reads mapped to the genome; unique mapped, the number of the reads mapped to a unique position in the genome; unique rate, the ratio of the reads mapped to a unique position in the genome.

**Table 3:** The most significant differentially expressed circular RNAs (circRNAs) (top 10 listed).

CircRNA_name	Chrome	Exon count	Gene name	logFC	logCPM	LR	*P* value	FDR
circ_POC1B_000154	chr12	6	POC1B	–5.905057	8.894108	13.44331	0.000246	0.277026
circ_RUFY2_003156	chr10	4	RUFY2	–5.727558	8.778988	11.1986	0.000819	0.691708
circ_HMGCR_002010	chr5	3	HMGCR	–5.434035	8.602139	8.488467	0.003574	0.711366
circ_NPLOC4_000576	chr17	3	NPLOC4	–5.243566	8.494641	7.161364	0.007449	0.711366
circ_CAPRIN2_000093	chr12	5	CAPRIN2	–5.228417	8.487116	7.073269	0.007824	0.711366
circ_ZMYND8_006470	chr20	4	ZMYND8	5.2603368	8.50343	7.25106	0.007086	0.711366
circ_SENP5_006766	chr3	1	SENP5	5.3402468	8.54897	7.790759	0.005251	0.711366
circ_ARFGEF1_008029	chr8	3	ARFGEF1	5.6240365	8.715481	10.05927	0.001516	0.711366
circ_RC3H2_007996	chr9	7	RC3H2	5.9666606	8.935713	14.32273	0.000154	0.260251
circ_DCBLD2_006709	chr3	5	DCBLD2	7.1049669	9.787842	39.81423	2.79E-10	9.44E-07

*Note.* FC, fold change; CPM, counts per million; LR, likelihood ratio; FDR, false discovery rate.

**Table 4:** The top 10 miRNAs targeting more circular RNAs (circRNAs).

MiRNA	Frequency
hsa-miR-4659b-3p	32
hsa-miR-4778-3p	32
hsa-miR-4659a-3p	30
hsa-miR-4691-5p	27
hsa-miR-6792-3p	27
hsa-miR-3059-5p	24
hsa-miR-6875-3p	24
hsa-miR-103a-1-5p	23
hsa-miR-103a-2-5p	22
hsa-miR-6881-3p	21

**Table 5:** The circular RNAs (circRNAs) correlated with non-small cell lung cancer.

CircRNA_name	Gene symbol	Disease name	Score	No. of Pmids	No. of Snps	Source
circ_MDM2_000139	MDM2	Non-small cell lung carcinoma	0.2085165	32	2	BEFREE; CTD_human
circ_ATF2_001418	ATF2	Non-small cell lung carcinoma	0.0005495	2	0	BEFREE
circ_DICER1_000834	DICER1	Non-small cell lung carcinoma	0.0008242	3	0	BEFREE
circ_PRKAA1_001969	PRKAA1	Non-small cell lung carcinoma	0.0002747	1	0	BEFREE
circ_BIRC6_001271	BIRC6	Non-small cell lung carcinoma	0.0002747	1	0	BEFREE
circ_BUB1_001363	BUB1	Non-small cell lung carcinoma	0.0002747	1	0	BEFREE
circ_RIPK1_001778	RIPK1	Non-small cell lung carcinoma	0.0005495	2	0	BEFREE
irc_CDC25C_002079	CDC25C	Non-small cell lung carcinoma	0.0002747	1	0	BEFREE

## Data Availability

The data used to support the findings of this study are available from the corresponding author upon request.

## Additional Points

*Highlights.* (1) There **were** 106 differentially expressed circRNAs between the XAV939-treated NSCLC cells and control. (2) ATF2 was enriched in the TNF signaling pathway. (3) Circ_**MDM2**_000139, circ_ATF2_001418, circ_CDC25C_002079, and circ_BIRC6_001271 were key circRNAs in XAV939-treated NSCLC cells.

## Supplementary Materials

Supplementary Materials The relative expression levels of key DE-circRNAs were detected by the real-time reverse transcription polymerase chain reaction (RT-PCR). The primer information is shown in the Supplementary Table 1.
