# Supplementary material for: Circ_MDM2_000139, Circ_ATF2_001418, Circ_CDC25C_002079, and Circ_BIRC6_001271 Are Involved in the Functions of XAV939 in Non-Small Cell Lung Cancer
Source: Can Respir J. 2019 Nov 27;2019:9107806. doi: 10.1155/2019/9107806 (PMC6900950; doi:10.1155/2019/9107806)
Supplement: Supplementary Materials — The relative expression levels of key DE-circRNAs were detected by the real-time reverse transcription polymerase chain reaction (RT-PCR). The primer information is shown in the Supplementary Table 1. [file 9107806.f1.pdf]

Supplementary Table 1 The primer sequences of key DE-circRNAs

primer	primer sequences (5’-3’)
circ_MDM2_000139-F	GAAGGGCAAGAGAGGTGGATCT
circ_MDM2_000139-R	CTTGGTCCTCGAGGCACTCAC
circ_ATF2_001418-F	GAACCCACATGCCTCTACCAA
circ_ATF2_001418-R	ATAGCCACTGACACGGGAACT
circ_DICER1_000834-F	GCTGGGTGTAGTGGTATGTGCT
circ_DICER1_000834-R	CCAGATCATCTCGGGCTCCTG
circ_PRKAA1_001969-F	CTGGAAGGACAACCGCACTG
circ_PRKAA1_001969-R	TGGACGAATAGGTGGTGAAACC

circ_RIPK1_001778-F	AATTCTCCGGCAGCTTACCC
circ_RIPK1_001778-R	GACAATCTGTGCCTGCTACGA
circ_CDC25C_002079-F	GCTGGTGACAATGCAGAATGCA
circ_CDC25C_002079-R	GGAACAGCCCCAGTTTCAATCAC
GAPDH-hF	TGACAACTTTGGTATCGTGGAAGG
GAPDH-hR	AGGCAGGGATGATGTTCTGGAGAG
